# Amyloglucosidase enzymatic reactivity inside lipid vesicles

**DOI:** 10.1186/1754-1611-1-4

**Published:** 2007-10-10

**Authors:** Mian Li, Michael J Hanford, Jin-Woo Kim, Tonya L Peeples

**Affiliations:** 1 Department of Chemical and Biochemical Engineering, University of Iowa, Iowa City, Iowa 52242, USA; 2 Department of Biological and Agricultural Engineering, University of Arkansas, Fayetteville, AR 72703, USA

## Abstract

Efficient functioning of enzymes inside liposomes would open new avenues for applications in biocatalysis and bioanalytical tools. In this study, the entrapment of amyloglucosidase (AMG) (EC 3.2.1.3) from *Aspergillus niger *into dipalmitoylphosphatidylcholine (DPPC) multilamellar vesicles (MLVs) and large unilamellar vesicles (LUVs) was investigated. Negative-stain, freeze-fracture, and cryo-transmission electron microscopy images verified vesicle formation in the presence of AMG. Vesicles with entrapped AMG were isolated from the solution by centrifugation, and vesicle lamellarity was identified using fluorescence laser confocal microscopy. The kinetics of starch hydrolysis by AMG was modeled for two different systems, free enzyme in aqueous solution and entrapped enzyme within vesicles in aqueous suspension. For the free enzyme system, intrinsic kinetics were described by a Michaelis-Menten kinetic model with product inhibition. The kinetic constants, *V*_*max *_and *K*_*m*_, were determined by initial velocity measurements, and *K*_*i *_was obtained by fitting the model to experimental data of glucose concentration-time curves. Predicted concentration-time curves using these kinetic constants were in good agreement with experimental measurements. In the case of the vesicles, the time-dependence of product (glucose) formation was experimentally determined and simulated by considering the kinetic behavior of the enzyme and the permeation of substrate into the vesicle. Experimental results demonstrated that entrapped enzymes were much more stable than free enyzme. The entrapped enzyme could be recycled with retention of 60% activity after 3 cycles. These methodologies can be useful in evaluating other liposomal catalysis operations.

## Background

Liposomes have long been used as carrier systems for the delivery of vaccines, therapeutic drugs and hormones because of easy preparation, good biocompatibility and biodegradability, low toxicity, and commercial availability [1-3]. Efficient functioning of enzymes inside liposomes opens up new possibilities of applications in biocatalysis and bioanalytical tools [4-6]. For example, enzyme-containing vesicles can serve as nanoreactors for biospecific reactions. In such reaction systems specific substrates, which permeate across the vesicle membrane lipid bilayer(s), are converted to products by the entrapped enzymatic catalyst [6,7].

The present work explores the development of novel reactive and stable biocatalytic interfaces for direct conversion of substrates. The reactivity of entrapped enzyme inside either multilamellar vesicles (MLV) or large unilamellar vesicles (LUV) liposomes was examined with respect to externally added substrate. The ultimate goal is to design stable catalytic interfaces that will mediate both chemical transformations and interphase transport for extended periods. The enzyme of choice for these experiments is Amyloglucosidase (E. C. 3. 2. 1. 3). As an industrial catalyst, amyloglucosidase (AMG) is one of the most economically important enzymes widely used in many industries such as baking, detergents, sewage treatment, and natural sweeteners [8]. This enzyme catalyzes both exo-(1–4) and branch-point (1–6)-linkages to produce glucose, providing the primary step in the conversion of agricultural feedstocks to ethanol [9]. However, after completion of each batch of reaction, the product is recovered using processes that denature the enzyme catalyst leading to loss of activity, thus increasing processing costs. To eliminate the disadvantages associated with the use of soluble AMG present in the conventional process, AMG has been immobilized on various carriers in an effort not only to retain catalytic activity for conventional processing, but also to maintain stability for repeated and continuous application [10-13]. Here, the utility of liposomal systems for enzyme stabilization and recycle is experimentally demonstrated, and mathematically described.

## Mathematical models

The kinetics of amyloglucosidase from different sources has been extensively investigated [14-16]. Process conditions, including temperature, pH, chain length of the starch, and starch concentration, have been found to influence the rate constants.

The rate of starch consumption by amyloglucosidase can generally be expressed in a Michaelis-Menten form with competitive product inhibition [15]:

−dSdt=Vmax⁡SKm(1+GKi)+S
 MathType@MTEF@5@5@+=feaafiart1ev1aaatCvAUfKttLearuWrP9MDH5MBPbIqV92AaeXatLxBI9gBaebbnrfifHhDYfgasaacH8akY=wiFfYdH8Gipec8Eeeu0xXdbba9frFj0=OqFfea0dXdd9vqai=hGuQ8kuc9pgc9s8qqaq=dirpe0xb9q8qiLsFr0=vr0=vr0dc8meaabaqaciaacaGaaeqabaqabeGadaaakeaacqGHsisldaWcaaqaaiabdsgaKjabdofatbqaaiabdsgaKjabdsha0baacqGH9aqpdaWcaaqaaiabdAfawnaaBaaaleaacyGGTbqBcqGGHbqycqGG4baEaeqaaOGaem4uamfabaGaem4saS0aaSbaaSqaaiabd2gaTbqabaGccqGGOaakcqaIXaqmcqGHRaWkdaWcaaqaaiabdEeahbqaaiabdUealnaaBaaaleaacqWGPbqAaeqaaaaakiabcMcaPiabgUcaRiabdofatbaaaaa@46E5@

where *S *is substrate concentration (mg mL^-1^), *V*_*max *_(mg mL^-1 ^min^-1^) the maximum rate of reaction, *K*_*m *_(mg mL^-1^) the Michaelis-Menten constant, *G *product concentration (mg ml^-1^), and *K*_*i *_(mg mL^-1^) the inhibition constant.

**Figure 1:**
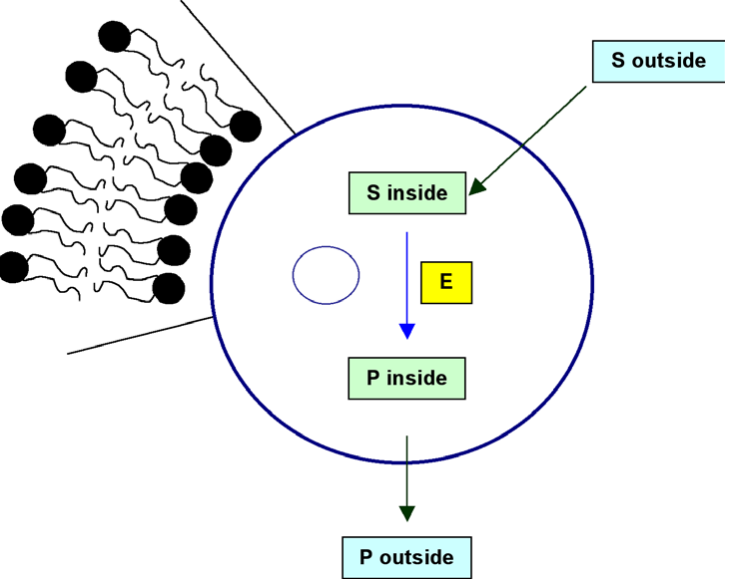
Schematic enzymatic hydrolysis inside a DPPC vesicle.

AMG-containing lipid vesicles were first proposed for use in enzyme-replacement therapy [17]. A quantitative understanding of enzyme reactions in vesicles is crucial to understanding the enzyme performance and mass transfer limitation [18]. An AMG-containing vesicle system is schematically illustrated in Figure 1. Starch diffuses from the bulk aqueous phase, permeates across the dipalmitoylphosphatidylcholine (DPPC) bilayer shell of the liposome into the vesicle's aqueous lumenal phase (interior), where the enzymatic hydrolysis reaction is catalyzed by entrapped AMG. The flux of starch from the external phase into the aqueous interior is assumed to follow Fick's first law of diffusion [19,20]. The mass balance for starch in the bulk solution is expressed in Equation 2.

d[S]outside,bulkdt=−kc([S]outside−[S]inside)
 MathType@MTEF@5@5@+=feaafiart1ev1aaatCvAUfKttLearuWrP9MDH5MBPbIqV92AaeXatLxBI9gBaebbnrfifHhDYfgasaacH8akY=wiFfYdH8Gipec8Eeeu0xXdbba9frFj0=OqFfea0dXdd9vqai=hGuQ8kuc9pgc9s8qqaq=dirpe0xb9q8qiLsFr0=vr0=vr0dc8meaabaqaciaacaGaaeqabaqabeGadaaakeaadaWcaaqaaiabdsgaKjabcUfaBjabdofatjabc2faDnaaBaaaleaacqWGVbWBcqWG1bqDcqWG0baDcqWGZbWCcqWGPbqAcqWGKbazcqWGLbqzcqGGSaalcqWGIbGycqWG1bqDcqWGSbaBcqWGRbWAaeqaaaGcbaGaemizaqMaemiDaqhaaiabg2da9iabgkHiTiabdUgaRnaaBaaaleaacqWGJbWyaeqaaOGaeiikaGIaei4waSLaem4uamLaeiyxa01aaSbaaSqaaiabd+gaVjabdwha1jabdsha0jabdohaZjabdMgaPjabdsgaKjabdwgaLbqabaGccqGHsislcqGGBbWwcqWGtbWucqGGDbqxdaWgaaWcbaGaemyAaKMaemOBa4Maem4CamNaemyAaKMaemizaqMaemyzaugabeaakiabcMcaPaaa@65F4@

where [*S*]_*outside *_and [*S*]_*inside*_are the substrate (starch) concentration outside and inside the vesicles at time t, respectively. *K*_*c *_is mass transfer coefficient. Once the vesicle surface area and volume are determined, permeability coefficient (*Ps*) for the substrate can be calculated [18].

The mass balance inside vesicle can be expressed in Equation 3.

d[S]insidedt=kc([S]outsde−[S]inside)−v
 MathType@MTEF@5@5@+=feaafiart1ev1aaatCvAUfKttLearuWrP9MDH5MBPbIqV92AaeXatLxBI9gBaebbnrfifHhDYfgasaacH8akY=wiFfYdH8Gipec8Eeeu0xXdbba9frFj0=OqFfea0dXdd9vqai=hGuQ8kuc9pgc9s8qqaq=dirpe0xb9q8qiLsFr0=vr0=vr0dc8meaabaqaciaacaGaaeqabaqabeGadaaakeaadaWcaaqaaiabdsgaKjabcUfaBjabdofatjabc2faDnaaBaaaleaacqWGPbqAcqWGUbGBcqWGZbWCcqWGPbqAcqWGKbazcqWGLbqzaeqaaaGcbaGaemizaqMaemiDaqhaaiabg2da9iabdUgaRnaaBaaaleaacqWGJbWyaeqaaOGaeiikaGIaei4waSLaem4uamLaeiyxa01aaSbaaSqaaiabd+gaVjabdwha1jabdsha0jabdohaZjabdsgaKjabdwgaLbqabaGccqGHsislcqGGBbWwcqWGtbWucqGGDbqxdaWgaaWcbaGaemyAaKMaemOBa4Maem4CamNaemyAaKMaemizaqMaemyzaugabeaakiabcMcaPiabgkHiTiabdAha2baa@5E23@

Michaelis-Menten kinetics with competitive product inhibition (Eq. 1) can be applied to describe the rate of enzymatic reaction (v) inside the vesicles (Eq. 4).

v=Vmax⁡[S]insideKm(1+[G]insideKi)+[S]inside
 MathType@MTEF@5@5@+=feaafiart1ev1aaatCvAUfKttLearuWrP9MDH5MBPbIqV92AaeXatLxBI9gBaebbnrfifHhDYfgasaacH8akY=wiFfYdH8Gipec8Eeeu0xXdbba9frFj0=OqFfea0dXdd9vqai=hGuQ8kuc9pgc9s8qqaq=dirpe0xb9q8qiLsFr0=vr0=vr0dc8meaabaqaciaacaGaaeqabaqabeGadaaakeaacqWG2bGDcqGH9aqpdaWcaaqaaiabdAfawnaaBaaaleaacyGGTbqBcqGGHbqycqGG4baEaeqaaOGaei4waSLaem4uamLaeiyxa01aaSbaaSqaaiabdMgaPjabd6gaUjabdohaZjabdMgaPjabdsgaKjabdwgaLbqabaaakeaacqWGlbWsdaWgaaWcbaGaemyBa0gabeaakiabcIcaOiabigdaXiabgUcaRmaalaaabaGaei4waSLaem4raCKaeiyxa01aaSbaaSqaaiabdMgaPjabd6gaUjabdohaZjabdMgaPjabdsgaKjabdwgaLbqabaaakeaacqWGlbWsdaWgaaWcbaGaemyAaKgabeaaaaGccqGGPaqkcqGHRaWkcqGGBbWwcqWGtbWucqGGDbqxdaWgaaWcbaGaemyAaKMaemOBa4Maem4CamNaemyAaKMaemizaqMaemyzaugabeaaaaaaaa@62BD@

## Methods

### Chemicals

AMG from *Aspergillus niger *(A-3042) and dipalmitoylphosphatidylcholine (DPPC) were purchased from Sigma (St. Louis, MO). Soluble starch was obtained from Aldrich (Milwaukee, WI). The fluorescent probe, 1,1'-dioctadecyl-3, 3,3', 3'-tetramethylindocarbocyanine perchlorate (DiIC_18 _(3)) was obtained from Molecular Probes (Eugene, OR). All other chemicals were of reagent grade and obtained from Fisher Scientific (Hanover Park, IL). All water used was purified using a Barnstead commercial deionization system (Boston, MA).

### Preparation of enzyme-containing liposomes

#### Multilamellar vesicles (MLVs)

MLVs were produced using the thin-film hydration method [21]. Various amounts of lipids (usually 4–22 mg DPPC) were dissolved into 10 ml of chloroform in a 100 ml round bottom flask. The solvent was removed to form a thin lipid film on the wall of the flask under reduced pressure using a rotary evaporator. Any residual solvent was removed either under vacuum overnight or under a stream of N_2_. AMG solution was prepared by diluting stock solution (Sigma A-3042) 10-fold with distilled deionized water. This solution (4 mL) was slowly added to the flask, followed by a small quantity of glass beads to provide mechanical agitation. The flask was returned to the rotary evaporator, immersed in a warm water bath and vigorously rotated to reconstitute the lipid film above the lipid's main transition temperature (T_c _= 41.3°C), approximately 5–10 minutes. A milky suspension of multilamellar vesicles (MLVs) was produced.

#### Large unilamellar vesicles (LUVs)

LUVs containing entrapped enzyme were produced via extrusion. In this process, AMG-containing MLV samples were centrifuged and suspended in AMG solution (0.1 X) to preserve the intravesicular enzyme concentration during extrusion. The suspension was passed 21 times through a stacked pair of polycarbonate filters (Avestin Inc., Ottawa, Canada) mounted in an extrustion device (Avestin, Lipo-fast), first with 1000 nm filters, then again using 200 nm filters [22]. A significant decrease in size and lamellarity can be achieved, resulting in a relatively monodisperse vesicles.

#### Giant unilamellar liposomes (GUVs)

In some experiments, giant unilamellar liposomes (GUVs) were prepared according to [23]. In brief, a desired quantity of DPPC was dissolved in 1 ml of chloroform and 200 μl of methanol. The aqueous phase containing 4 mL AMG was then carefully added along the flask walls. The organic solvent was removed in a rotary evaporator. After evaporation for 2 min, an opalescent fluid was obtained. The resulting aqueous solution contained GUVs.

### Separation of the free and entrapped enzymes

Liposomal suspensions were centrifuged at 16 000 g for 30 min at 4°C to separate unencapsulated AMG from liposomes. The supernatant was removed and 4 mL water was added to the pellet to resuspend the liposomes before repeating the centrifugation. This wash process was repeated three times to ensure complete removal of the free enzyme from the liposome preparations. Free enzyme removal was verified by total protein assay and starch hydrolysis activity, the details of which are described in the later section, for each supernatant. After the final centrifugation, the pellet was resuspended in 2 mL water prior to determination of entrapped protein and starch hydrolysis activity.

### Determination of entrapment percent and entrapment efficiency

Entrapment percent and entrapment efficiency were determined after removal of external enzyme by repeated centrifugation and washing. The entrapment percent (EP) and entrapment efficiency (EF) can be calculated by subtracting the amount of free enzyme in the supernatant from the total amount of the added enzyme as:

EP=total enzyme−free enzymetotal enzyme×100%
 MathType@MTEF@5@5@+=feaafiart1ev1aaatCvAUfKttLearuWrP9MDH5MBPbIqV92AaeXatLxBI9gBaebbnrfifHhDYfgasaacH8akY=wiFfYdH8Gipec8Eeeu0xXdbba9frFj0=OqFfea0dXdd9vqai=hGuQ8kuc9pgc9s8qqaq=dirpe0xb9q8qiLsFr0=vr0=vr0dc8meaabaqaciaacaGaaeqabaqabeGadaaakeaacqWGfbqrcqWGqbaucqGH9aqpdaWcaaqaaiabbsha0jabb+gaVjabbsha0jabbggaHjabbYgaSjabbccaGiabbwgaLjabb6gaUjabbQha6jabbMha5jabb2gaTjabbwgaLjabgkHiTiabbAgaMjabbkhaYjabbwgaLjabbwgaLjabbccaGiabbwgaLjabb6gaUjabbQha6jabbMha5jabb2gaTjabbwgaLbqaaiabbsha0jabb+gaVjabbsha0jabbggaHjabbYgaSjabbccaGiabbwgaLjabb6gaUjabbQha6jabbMha5jabb2gaTjabbwgaLbaacqGHxdaTcqaIXaqmcqaIWaamcqaIWaamcqGGLaqjaaa@6539@

EF=total enzyme−free enzymeamount of phospholipid used
 MathType@MTEF@5@5@+=feaafiart1ev1aaatCvAUfKttLearuWrP9MDH5MBPbIqV92AaeXatLxBI9gBaebbnrfifHhDYfgasaacH8akY=wiFfYdH8Gipec8Eeeu0xXdbba9frFj0=OqFfea0dXdd9vqai=hGuQ8kuc9pgc9s8qqaq=dirpe0xb9q8qiLsFr0=vr0=vr0dc8meaabaqaciaacaGaaeqabaqabeGadaaakeaacqWGfbqrcqWGgbGrcqGH9aqpdaWcaaqaaiabbsha0jabb+gaVjabbsha0jabbggaHjabbYgaSjabbccaGiabbwgaLjabb6gaUjabbQha6jabbMha5jabb2gaTjabbwgaLjabgkHiTiabbAgaMjabbkhaYjabbwgaLjabbwgaLjabbccaGiabbwgaLjabb6gaUjabbQha6jabbMha5jabb2gaTjabbwgaLbqaaiabbggaHjabb2gaTjabb+gaVjabbwha1jabb6gaUjabbsha0jabbccaGiabb+gaVjabbAgaMjabbccaGiabbchaWjabbIgaOjabb+gaVjabbohaZjabbchaWjabbIgaOjabb+gaVjabbYgaSjabbMgaPjabbchaWjabbMgaPjabbsgaKjabbccaGiabbwha1jabbohaZjabbwgaLjabbsgaKbaaaaa@72BB@

### Characterization of enzyme-containing liposomes

#### Negative staining electron microscopy

A drop of freshly prepared liposome was applied to a Formvar-coated copper grid. After 20 seconds, the excess liquid was absorbed at the periphery of the grid by filter paper. The remaining sample was air-dried at room temperature and the liposome was negatively stained with 0.5% uranyl acetate or ammonium molybdate for 20 s, after which most of the staining solution was absorbed at the periphery of the grid by means of filter paper. Samples were examined with a Hitachi H-600 transmission electron microscope (TEM).

#### Freeze-fracture and electron microscopy

A small aliquot of a liposome sample was placed between two copper strips, a double wing replica holder. The holder was plunged into liquid propane (-190°C) for approximately 10 seconds. Frozen samples were inserted into a hinged double replica device and transferred into a Balzer 301 freeze-fracture apparatus. Fracturing was performed at -120°C and about 10^-7 ^torr by releasing a spring that opened the two sides of the replica holder. Samples were immediately replicated with platinum and coated with carbon. Replicas were cleaned in an acidic mixture of nitric acid, sulfuric acid and acetic acid and washed with distilled water. The clean replicas were collected onto uncoated 400 mesh electron microscope copper grids and were examined with a Hitachi H-600 TEM.

#### Cryo-transmission electron microscopy (cryo-TEM)

A drop of liposome sample was applied to a standard electron microscopy grid coated with a perforated carbon film. Excess liquid was removed by blotting with filter paper, leaving a thin layer of aqueous sample covering the holes of the carbon film. The grid was rapidly frozen in liquid ethane, resulting in vesicles embedded in a thin film of vitreous ice. Images of the vesicles in ice were obtained under cryogenic conditions using a Gatan cryo-holder in a Hitachi H-600 TEM and a defocus of -1.5 μm.

#### Confocal microscopy

Fluorescent probe (DiIC_18_(3)) was added to the lipid at a concentration of 0.1 mol %. Confocal images were obtained with a MRC 1024 confocal microscope (Bio-Rad) with a 585 LB emmision filter at 488 nm excitation. For three-dimensional image projection of a vesicle, z-scans in 0.5 μm steps were taken through the upper half of a liposome and projected by using the Confocal Assistant 4.02 software.

### Hydrolytic activity and protein assays

AMG activity was determined by the starch-iodine method as described in [24]. The assay solution consisted of 0.1 mL enzyme-containing sample and 1.0 mL starch solution (1%, w/v) in distilled water at pH 4.5. Assay tubes were incubated for 4 min at 55°C in a dry bath incubator. Appropriate negative controls, samples prepared without enzyme were made in all cases. One unit of enzyme activity was defined as the amount of enzyme, which hydrolyzed 1 mg of starch per minute under specified conditions.

Protein content of enzyme solution was determined using the Bio-Rad (Richmond, CA) *DC *Protein Assay kit with bovine serum albumin as a standard.

Sugars produced by the enzymatic hydrolysis of starch were identified and quantified by a Shimadzu HPLC system (Liquid Chromatograph LC-10AT, Diode Array SPD-M10A, and RID 6A) equipped with an Aminex HPX-87H cation-exchange column (300 mm × 7.8 mm, Bio-Rad Laboratories, Richmond, CA). The column was maintained at 50°C using a Bio-Rad column heater. Samples were eluted isocratically with 5 mM H_2_SO_4 _at a flow rate of 0.4 ml min^-1^. Maltooligosaccharides were purchased from Sigma Chemical Co. (St. Louis, MO) and used as standards as described previously [24].

### Intrinsic enzyme kinetics

Experiments for kinetic parameter estimation were performed by hydrolyzing starch solutions of varying concentrations (1 – 10 mg mL^-1^) at 55°C in a dry bath incubator. The reaction medium consisted of 0.1 mL free or entrapped AMG, and 1.0 mL starch solution. Starch solutions were prepared in distilled, deionized H_2_O (pH 4.5). For each specified concentration, a series of identical reaction tubes containing starch solution was prepared. Incubation of the tubes was initiated at the same time. Samples, consisting of one reaction tube per time point, were taken at 0.5 min intervals for 10 min. The reaction in a tube was stopped by adding 1 mL HCl (1.0 M). The amount of unreacted starch was estimated by the starch-iodine assay, and glucose concentrations by HPLC as described above. Each experiment was conducted in triplicate. Initial rates were calculated using the linear portion of substrate vs. time plot.

### Starch hydrolysis by entrapped AMG

Starch hydrolysis was carried out in liposomal suspensions. Soluble starch was used as substrate, and the process conditions were 55°C and pH 4.5 prior to the addition of entrapped AMG. Samples (1 mL) were taken at 10–60 min intervals, and the reaction was stopped by quenching in ice and adding 1.0 mL HCl (1.0 M). Samples were further analyzed by HPLC as described above.

### Repeated hydrolysis of starch

The capacity for recovery and recycle of MLV-entrapped AMG activity from a process stream was also determined. The rate of starch hydrolysis by entrapped enzyme was determined at 55°C in 1.0 % (w/v) starch. After each batch of hydrolysis, the entrapped enzyme preparations were recovered from the reaction mixture by centrifugation at 16000 g for 10 min. The pellet was then resuspended into fresh starch solution for the subsequent run of starch hydrolysis. The whole process was repeated for six runs.

### Numerical analyses

Kinetic parameters for starch hydrolysis were estimated using the nonlinear parameter estimation software of TableCurve 2D (Jandel Scientific, San Rafael, CA) based on initial rate data. Polymath 5.0 [25] (Special Education Version, Prentice Hall, New Jersey) was used to solve the ordinary differential equations. The programs gave concentration profiles of substrate (starch) and product (glucose) for each set of conditions evaluated.

## Results and discussion

### Physical characterization of liposomes

**Figure 2:**
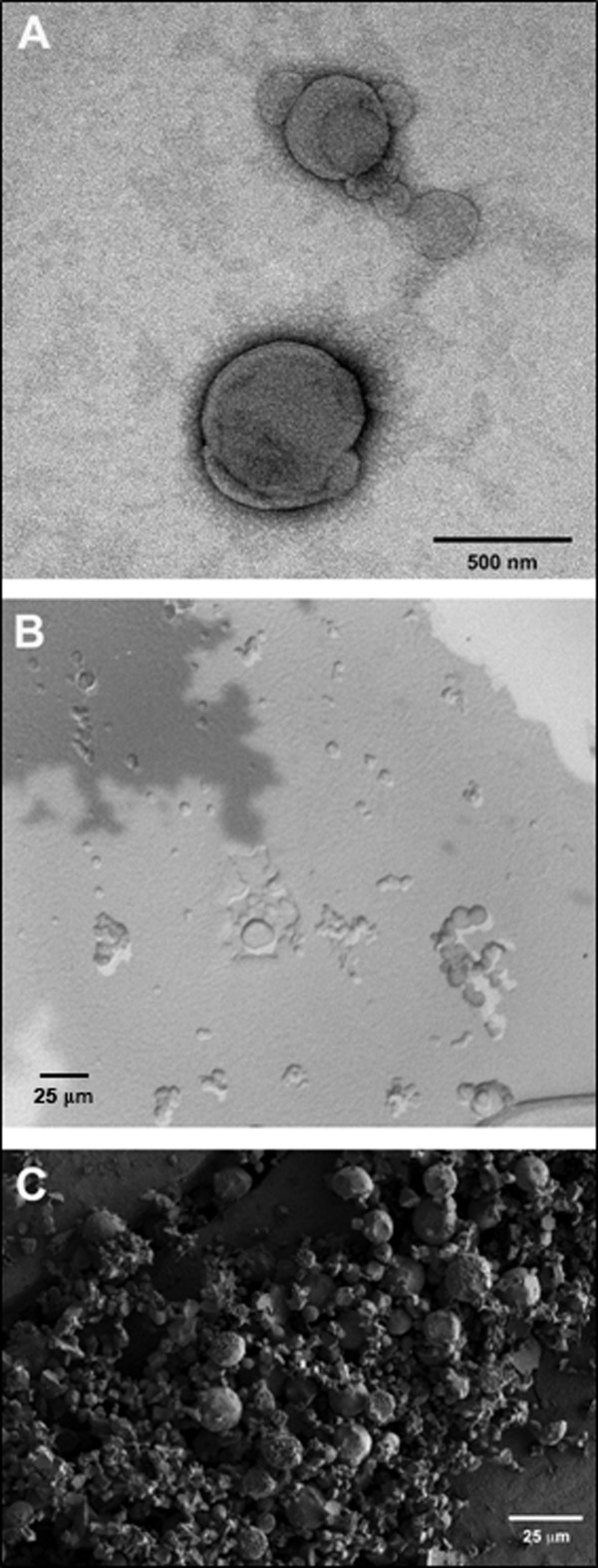
Representative micrograph of DPPC MLVs. A: Negative-staining image. B: Freeze-fracture TEM. C: Cryo-SEM image.

Structural details were visualized by negative-staining, freeze-fracture and cryo-TEM. Figure 2 shows a representative micrograph of a DPPC MLV containing AMG prepared by the thin film hydration method: a negative-staining image of a sample with an entrapped enzyme-to-lipid ratio of 0.349 mg/mg (Fig. 2A), a freeze-fracture TEM (Fig. 2B), and a cryo-TEM image (Fig. 2C). Cumulative data obtained by these techniques showed that the vesicles are spherical in shape with a wide size distribution ranging from approximately 0.2 to 10 *μm*. These results may indicate that aggregation of liposomes upon entrapping the enzyme is occurring.

**Figure 3:**
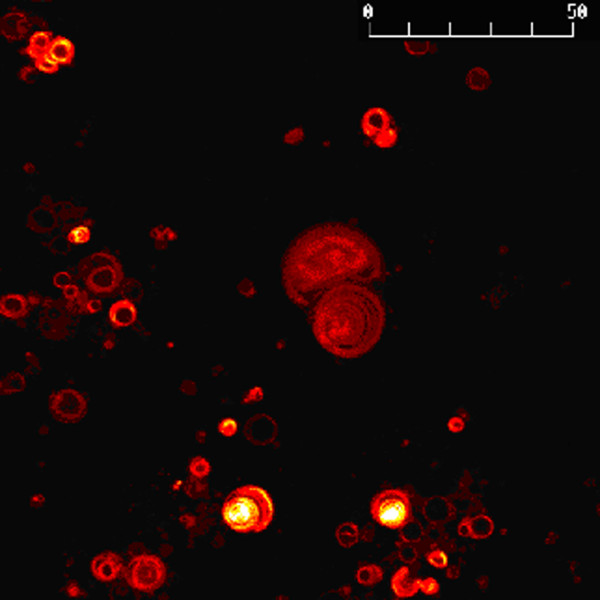
Representative micrograph of DPPC MLVs by confocal microscopy. The unit of the scale bar is μm.

Typically, freeze fracture electron microscopy is an effective method to ascertain vesicle lamellarity. Despite repeated attempts, bisecting cleavage of the vesicles to reveal lamellar structure was not observed in our samples. Therefore, we explored the capacity to use confocal microscopy to demonstrate the lamellar multiplicity. The confocal image in Figure 3 clearly illustrates the presence of multi-lamellar vesicles in samples produced by the thin-film rehydration methods.

### Entrapment study

Entrapment studies of AMG in liposomal preparations were initiated by incorporation of AMG into the DPPC MLVs. To test the reactivity of entrapped AMG, it was important to ensure that no extravesicular enzyme was present in our preparations. The removal of entrained AMG from the liposome preparations was complete, as less than 0.05% total protein was detected in the final supernatant of the wash preparation.

Table 1 lists AMG entrapment percentage and entrapment efficiency determined after removal of external enzymes by successive centrifugation, washing and re-dispersion steps. Enzyme entrapment is directly proportional to DPPC concentration. However, the entrapment efficiency decreases with increasing DPPC content. The lower entrapment efficiency suggests a disproportionately low increase in capture volume. That is, the addition of more lipid increases the lamellarity of the vesicle population rather than producing more vesicles of the same lamellarity. This is likely an artifact of the thin film rehydration method. As the DPPC concentration increases, the thickness of the lipid film deposited on a round bottom flask wall is likely to increase, resulting in more vesicles of higher lamellarity. With more concentric bilayer shells, the MLVs will have a lower capture volume than MLVs prepared with lesser amounts of DPPC (thinner DPPC film), provided that size distribution, bilayer thickness and interlamellar spaces of these MLVs are constant.

**Table 1:** AMG entrapment in DPPC MLVs

DPPC concentration (mg/ml)	Entrapment percent (%)	Entrapment Efficiency (mg protein/mg DPPC)	Activity of entrapped AMG (Unit/mg protein)
2	6.83	0.507	8.86
4	9.35	0.349	5.76
7.8	13.17	0.252	5.84
11.25	14.45	0.188	6.68

The apparent activity of entrapped AMG was measured as shown in Table 1. It would be expected that the more protein is entrapped, the more activity appears. However, as entrapment percent is increased by 2-fold from 6.83% to 14.45%, the apparent activity decreases from 8.86 units/mg protein to 6.68 units/mg protein. The lower apparent activity may be due to mass transfer limitation.

**Figure 4:**
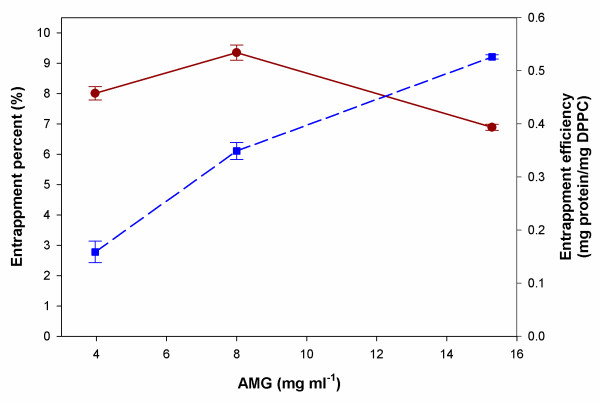
The entrapment percentage and efficiency as a function of AMG concentration. Filled circle (red circle): Entrapment percent. Filled square (blue square): Entrapment efficiency.

The entrapment percentage and efficiency as a function of AMG concentration at a constant DPPC concentration content was also investigated. Maximum entrapment percent was reached at 7.8 mg/ml AMG as shown in Figure 4. In addition, an increase in the entrapment efficiency with an increasing AMG concentration was observed.

### Thermostability of AMG entrapped in MLV and GUV

**Figure 5:**
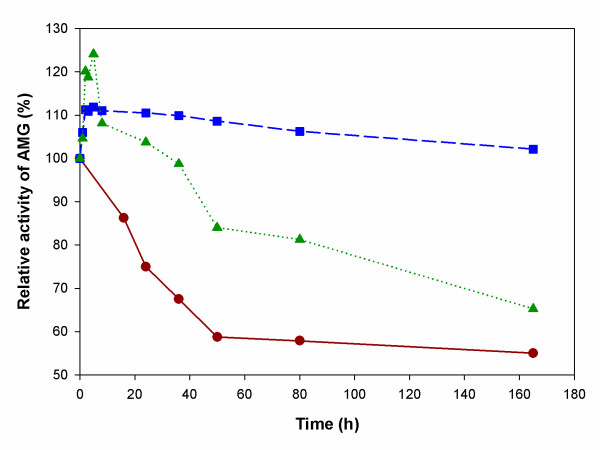
Thermostability of AMG. Filled circle (red circle): Soluble AMG. Filled square (blue square): MLVs. Filled triangle (green triangle): GUVs. Experiments were carried out at pH 5.0 and temperature 50°C. The initial activities were taken as 100%.

To evaluate the thermostability of free and entrapped AMG, the enzyme solution was incubated at the hydrolysis temperature in sealed tubes. Samples were taken after various incubation intervals, the residual enzyme activity was determined, and the relative enzyme activity was estimated by assuming the initial enzyme activity as 100%. Figure 5 shows the thermostability of MLV and GUV samples compared to the free AMG sample on the basis of the estimated relative AMG activities in each sample. AMG entrapped inside MLV and GUV remains preserved for a much longer period of time in comparison to the activity of the free enzyme in aqueous media. At 55°C, the native enzyme retained 55 % activity after 160 h whereas the entrapped enzyme retained 70 % and 100 % activity under identical conditions for GUV and MLV, respectively. Both the GUV and MLV studies show an increase of starch hydrolysis activity within the first few hours of high temperature incubation. This may reflect a change in the state of the entrapped enzyme over time or a change in liposome properties (which would impact starch permeability) at the hydrolysis temperature. Further study is needed to elucidate this phenomenon.

### Kinetic parameter estimation

**Figure 6:**
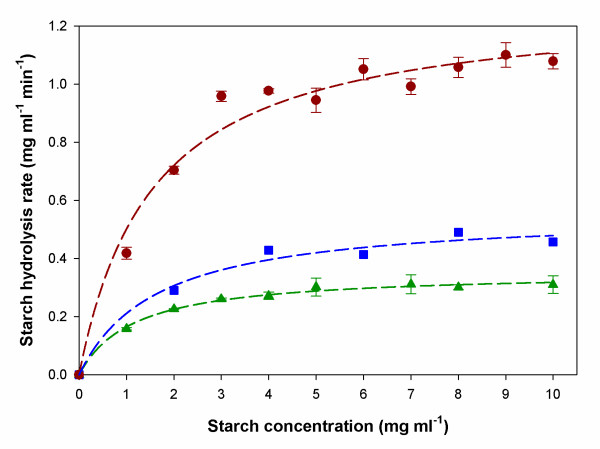
Initial rate study for entrapped AMG in DPPC. Filled circle (red circle): Soluble AMG. Filled square (blue square): LUVs. Filled triangle (green triangle): MLVs.

Kinetic constants (*K*_*m *_and *V*_*max*_) were determined from a classic enzyme kinetic analysis based on initial velocity measurements of soluble starch hydrolysis by either free AMG or entrapped AMG in MLV or LUV at various starch concentrations (1 – 10 mg mL^-1^). Results from this analysis are plotted in Figure 6. *K*_*i *_was determined by fitting experimental product concentration versus time data to the Michaelis-Menten equation. Values for the kinetic parameters are summarized in Table 2. The best-fit kinetic parameters of the Michaelis-Menten model were estimated by non-linear regression as *V*_*max *_= 1.28 mg glucose ml^-1 ^min^-1 ^mg^-1 ^protein and *K*_*m *_= 1.55 mg/ml, *V*_*max *_= 0.35 mg glucose ml^-1 ^min^-1 ^mg^-1 ^protein and *K*_*m *_= 1.15 mg/ml, *V*_*max *_= 0.56 mg glucose ml^-1 ^min^-1 ^mg^-1 ^protein and *K*_*m *_= 1.64 mg/ml, for free AMG, MLV and LUV, respectively. It should be pointed out that kinetic parameters for free AMG are intrinsic while those measured with MLVs and LUVs are apparent values due to mass transfer effects. Consistent with this, values of *V*_*max *_were significantly lower in the entrapped samples for both MLVs and LUVs compared to that of the free AMG. This is in agreement with literature data reported for amylase entrapped in soybean phosphatidylcholine liposomes [26]. The decrease in *V*_*max *_can be attributed to steric effects resulting from limitation of the accessibility of soluble substrate to the active site. Since MLVs have more lamellarity than LUVs, LUV has a larger value of *V*_*max *_(0.56) than that of MLVs (0.35) due to lower mass transfer limitations. The apparent *K*_*m *_values for MLV-entrapped AMG was lower than that for the free enzyme. The apparent *K*_*m *_for GUV-entrapped enzyme was not significantly different from the free enzyme *K*_*m*_. The glucose inhibition constant (*K*_*i*_) was determined to be 0.10 mg mL^-1 ^for all cases. While it is reasonable to believe that the apparent values differ from the free enzyme K_m _and V_max _because of the added transport layer(s) in the liposomes, with the data presented, the possibility that the intrinsic enzyme kinetics could be altered in the case of liposome entrapment cannot be completely excluded. The lower apparent V_max _for the multi-layer liposome relative to the single layer is most likely a reflection of the increased mass transfer barrier resulting form the additional transport layers. This provides additional evidence that liposomal kinetics is strongly a function of mass transfer.

**Table 2:** Kinetic parameters for single and dual enzyme systems.

	V_max_ (mg mL^-1^min^-1^)	K_m_ (mg mL^-1^)	K_i_ (mg mL^-1^)
Free enzyme (intrinsic)	1.28 ± 0.06	1.55 ± 0.29	0.10 ± 0.01
MLV (apparent)	0.35 ± 0.01	1.15 ± 0.12	0.10 ± 0.01
LUV (apparent)	0.56 ± 0.04	1.64 ± 0.25	0.10 ± 0.01

### Simulation of batch starch hydrolysis

**Figure 7:**
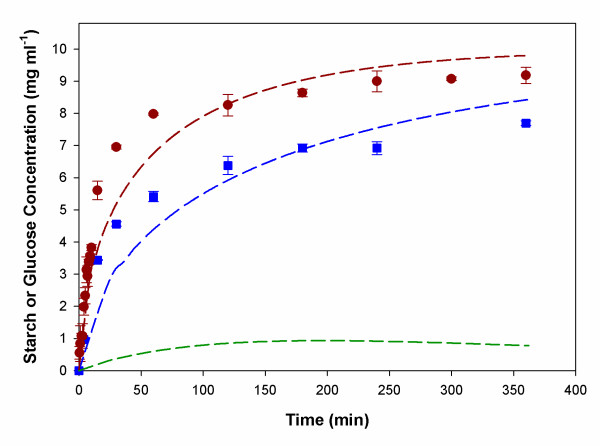
Model simulation and experimental validation. Red (soluble AMG) and blue (MLV) lines are simulation results of glucose concentration profiles. Symbols are experimental results of the glucose concentration profiles: Soluble AMG (filled red circle) and MLV (filled blue square). The green line is a simulation result of starch concentration profile inside MLV.

Enzymatic starch hydrolysis by free AMG and entrapped AMG into MLV were investigated for reaction systems containing 1% soluble starch. Glucose concentration was measured versus time. The difference of intrinsic and apparent kinetics was due to the substrate permeability across the bilayer membrane such that the experimental data were used to fit a mass transfer coefficient, by using Equations 2–4. Figure 7 shows the glucose concentration profiles (filled circle and filled square) compared with the simulated results (red and blue lines). The model simulations were in fair agreement with experimental data. In addition, the model and parameter estimation procedure allowed not only the quantification of the substrate permeability in the vesicle system used, but also provided insight into the changes of substrate concentrations inside the vesicles (green line), which would be rather difficult to determine experimentally.

### Repeated hydrolysis of starch

**Figure 8:**
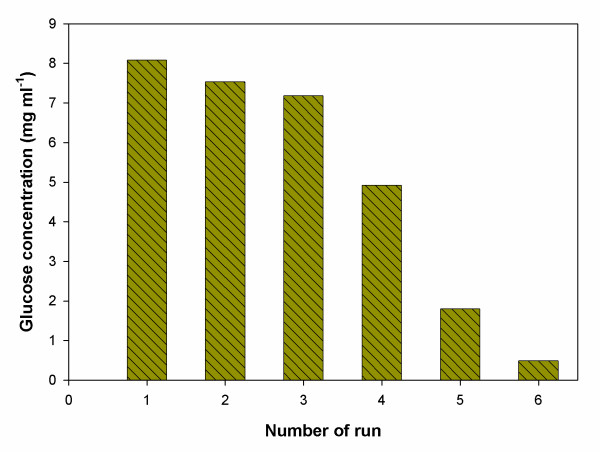
Repeated runs using MLVs for enzymatic hydrolysis of starch.

Figure 8 shows AMG activity in the repeated enzymatic hydrolysis of starch with vesicle-entrapped enzyme. Enzyme activity is very stable during the first three batch runs. The AMG lost 39.1% of original activity after the fourth batch run (after 96 hrs in processing). Beyond cycle four a great decrease in the degree of hydrolysis was observed. The decrease could be due to either vesicle leakage, loss of enzyme by adsorption to the substrates, or incomplete precipitation. The fact that enzymes are recycled in the process will make an improved process economy possible.

Compared to free AMG, the rate of hydrolysis by entrapped AMG is relatively low. This is either because of the low permeability of substrate across the liposome bilayer or because of the low enzyme activity inside the liposomes. In the current work, while apparent values for K_m _and V_m _are presented, no attempt was made to determine if transport across the lipid layer is rate-limiting, or if a lower reaction rate due to altered enzyme kinetics gives comparable time constants to mass transfer rates. Generally Thiele modulus, φ, is used for this purpose. For example, when φ is sufficiently small (φ < 0.3), diffusion of substrate is fast relative to its consumption. But in most cases, slow permeation of external substrate results in the low overall reaction rate. Currently there are generally two approaches to increase the substrate permeability. One is to reconstitute membrane channel proteins in the liposome bilayers while the other is to utilize lipid/detergent hybrid membranes [27].

## Conclusion

The kinetics of starch hydrolysis in the AMG-liposome microreactor system was characterized experimentally and mathematically. This methodology has the potential to be applied to evaluate other liposomal catalysis operations. In addition to investigating AMG entrapment percentage and entrapment efficiency, enhanced thermostability of liposome-entrapped AMG was demonstrated. As expected, hydrolysis rates are limited by the rate of mass transfer of substrate across the lipid bilayer. Activation of enzymes and altered permeability within the liposomal environment are being investigated further. The application of fluorescence laser confocal microscopy in physical characterization of liposome lamellarity was a useful methodology. Multiple batch hydrolyses of starch with entrapped AMG inside MLV demonstrated that MLV-entrapment was useful for catalysis with enzyme recovery. Such recycle operations reduce the cost of AMG required. These results show the promise of liposomes as enzyme carriers in conversions involving macromolecular reactants. Thus, the information gained from this research should contribute to improving our ability to advance biologically based processes by providing efficient and economical ways of enhancing the activity and stability as well as reusability of biocatalysts for use in bioprocessing applications.

## List of abbreviations used

*EF *entrapment efficiency

*EP *entrapment percent

*k*_*c *_mass transfer coefficient

*K*_*i *_the inhibition constant (mg mL^-1^)

*K*_*m *_the Michaelis-Menten constant (mg mL^-1^)

*P *product concentration (mg mL^-1^)

φ thiele modulus

*S *substrate concentration (mg mL^-1^)

*S*_*inside *_substrate concentration (mg mL^-1^) in the liposome phase

*S*_*outside *_substrate concentration (mg mL^-1^) in the bulk phase

*V*_*max *_maximum rate of reaction (mg mL^-1 ^min^-1^)

v rate of enzymatic reaction (mg mL^-1 ^min^-1^)

## Competing interests

The author(s) declare that they have no competing interests.

## Authors' contributions

ML and MJH contributed to the design of the study, the acquisition and analysis of data, and the writing of the manuscript. JWK and TLP contributed to the design and coordination of the study and participated in the writing of the manuscript. All authors read and approved the final manuscript.
